# Combining Ozonated Autohemotherapy with Pharmacological Therapy for Comorbid Insomnia and Myofascial Pain Syndrome: A Prospective Randomized Controlled Study

**DOI:** 10.1155/2022/3562191

**Published:** 2022-11-23

**Authors:** Wang Shen, Ning Liu, Zhonghua Ji, Hongwei Fang, Feng Liu, Wei Zhang, Xiuqin Yu, Mingxia Wang, Jinyuan Zhang, Xiangrui Wang

**Affiliations:** ^1^Department of Pain, Shanghai East Hospital Affiliated Tongji University, Shanghai, China; ^2^Department of Anesthesiology, Shanghai East Hospital Affiliated Tongji University, Shanghai, China; ^3^Department of Anesthesiology, Zhongshan Hospital Affiliated Fudan University, Shanghai, China

## Abstract

**Objective:**

To examine the efficacy and safety of ozonated autohemotherapy (O3-AHT) combined with pharmacological therapy for comorbid insomnia and myofascial pain syndrome (MPS).

**Materials and Methods:**

One hundred and eighteen patients were randomly divided into two groups: the control group (*N* = 50) and the O_3_-AHT group (*N* = 53). Patients in both groups were given the same pharmacological management for three weeks. Patients in the O_3_-AHT group were treated with ozonated autohemotherapy (the concentration of ozone was 20 *µ*g/ml in the first week, 30 *µ*g/ml in the second week, and 40 *µ*g/ml in the third week) combined with pharmacological therapy. Primary (the insomnia severity index (ISI) and visual analogue scale (VAS)) and secondary outcomes (the Epworth sleepiness scale (ESS), polysomnography data, the anxiety and preoccupation about sleep questionnaire (APSQ), the beck depression index (BDI), and the multidimensional fatigue inventory (MFI)) were examined at pretreatment, posttreatment, 1 month, and 6 months.

**Results:**

Fifty patients in the control group and fifty-three patients in the O_3_-AHT group completed the study. In both groups, insomnia and pain symptoms were relieved significantly compared with pretreatment. Compared with the control group, the O_3_-AHT group had significantly improved sleep quality, pain, and negative mood at different time points. No adverse complications were observed in either group.

**Conclusion:**

Compared with pharmacological therapy alone, ozonated autohemotherapy combined with pharmacological therapy can ameliorate insomnia, reduce pain intensity, improve negative mood, and alleviate fatigue more effectively without serious adverse complications.

## 1. Introduction

Myofascial pain syndrome (MPS), defined as a kind of painful and aseptic skeletal muscle inflammation, is one of the main causes of chronic pain in clinics. This complicated syndrome is characterized by myofascial trigger points (MTrPs) within fascia and muscles, and pressing on MTrPs can induce localized and referred pain [1]. MPS is a common disease faced by pain physicians in clinical practice, with a prevalence of about 30%–85% in patients with musculoskeletal pain [2].

Insomnia, defined as at least 3 months of difficulty in initiating and/or maintaining restorative sleep, is a common and debilitating comorbidity of musculoskeletal painful diseases [3]. The comorbidity of insomnia and pain is a fairly destructive situation for patients, and there is a complex bidirectional connection between insomnia and pain [4]. They aggravate each other reciprocally or even generate a vicious circle wherein pain disrupts sleep rhythms and poor sleep lowers the pain perception threshold in turn [5]. Although the bidirectional relationship between insomnia and pain has been extensively acknowledged, little scientific knowledge about the fundamental mechanisms underlying this complicated association has been entrenched. Recent researches consider that the neurochemical mechanisms involve immune-related inflammatory reaction and multiple neurohumoral regulations including the opioid system, monoaminergic system, pineal melatonin system, nitric oxide signaling, etc [6]. Comorbid insomnia and pain affect the life quality of patients substantially, causing symptoms such as daytime fatigue, emotional distress, and various dysfunctions. These complexities make the management of comorbid insomnia and chronic pain difficult.

The close relationship between insomnia and pain suggests the importance of effectively managing patients' sleep when treating patients with MPS. However, in clinical practice, pain physicians often focus on improving pain symptoms and ignore the potential harm caused by insomnia. The overall goals of treating patients with comorbid insomnia and MPS are improving sleep quality, alleviating pain, relieving negative emotions, and reducing the risk of insomnia-related disorders. Current strategies for the management of insomnia include cognitive behavioral therapy, medication, acupuncture, and other complementary therapies [7]. Cognitive behavioral therapy for insomnia (CBT-I), which is usually recommended as the first choice for the treatment of insomnia, has been reported to be effective in improving the symptoms of patients with comorbid insomnia and chronic pain as well [8]. CBT-I was generally considered to have few adverse complications compared with medication, but it has been reported that sleep restriction therapy, a standard behavioral strategy used within CBT-I, is associated with reduced objective total sleep time, increased daytime sleepiness, and objective performance impairment [9]. Pharmacological agents for insomnia include benzodiazepines such as alprazolam, nonbenzodiazepines such as zolpidem, and several antipsychotics [7]. Pharmacological agents for MPS include nonsteroidal anti-inflammatory drugs (NSAIDs), tricyclic antidepressants (TCAs), and muscle relaxants [1]. Medication may cause adverse effects such as gastrointestinal bleeding, renal injury, vomiting, and dizziness. Therefore, it is necessary to find an effective, safe, and feasible treatment for comorbid insomnia and MPS.

Recently, a multitude of studies has focused on the therapeutic value of ozone. In general, ozone's mechanism of action can be summarized as antioxidant capacity, vascular and hematological modulation, pathogen inactivation, and immune system activation [10]. Ozonated autohemotherapy (O_3_-AHT) is a common pattern of ozone therapies during which autologous blood is ozonated and then injected back into the body [11]. Recently, several studies have investigated the effect of O_3_-AHT on coronavirus 19 [12]. Besides, O_3_-AHT has shown significant advantages in diverse diseases such as psoriasis, age-related macular degeneration, and multiple sclerosis [13–15]. In terms of painful diseases, O_3_-AHT has been reported to relieve pain in fibromyalgia and postherpetic neuralgia [16, 17]. In addition, a study using low-dose ozone to treat insomnia in patients with coronary heart disease found that low-dose ozone therapy improved sleep quality and ameliorated depression by elevating the levels of brain-derived neurotrophic factor (BDNF) in blood serum [18].

As mentioned above, there is a complicated correlation between insomnia and pain, which poses a knotty challenge for management. We hypothesized that combining O_3_-AHT with pharmacological therapy can provide safe and effective relief of insomnia and pain in patients with comorbid insomnia and MPS. Therefore, we conducted a prospective, randomized, controlled study to evaluate the efficacy and safety of this combination therapy.

## 2. Materials and Methods

### 2.1. Ethics and Patients

This study was approved by the Institutional Review Board and Ethics Committee of Shanghai East Hospital (ChiCTR1900021602). The present clinical research was conducted in accordance with the Declaration of Helsinki. All authors had access to the study data and approved the manuscript. Written informed consent was obtained from all patients before the inclusion. From March 2019 to October 2021, we invited 176 patients to participate in our study on the basis of inclusion and exclusion criteria. The inclusion criteria are as follows: (1) age >18 years; (2) suffering from back pain for at least 3 months; (3) underwent a pain physician interview and a thorough physical examination to confirm the MPS diagnosis [19]; (4) diagnosed with insomnia according to the International Classification of Sleep Disorders, Third Edition (ICSD-3); and (5) willing to undergo randomization and follow instructions. The exclusion criteria are as follows: (1) the presence of other sleep diseases such as sleep apnea and restless legs syndrome; (2) unstable psychiatric disorders such as bipolar disorder, mania, and schizophrenia; (3) severe system disorders such as heart failure, liver dysfunction, renal injury, hematologic disorders, and coagulation dysfunction; (4) abuse of psychotropic substances or analgesics; (5) suffering from other painful diseases, especially cancer pain; (6) participating in other psychological treatments and/or drug trials; (7) being allergic to anticoagulants such as sodium citrate; and (8) being pregnant or lactating women. All patients were randomly divided into the O_3_-AHT group or the control group using a computerized number generator.

### 2.2. Sample Size

Because there was no reference for the effectiveness of ozonated autohemotherapy in patients with comorbid chronic insomnia and MPS, we conducted a preliminary trial before conducting the formal research. The preliminary trial indicated that the effective rate of treatment was 45% (9/20) in the control group and 75% (15/20) in the O_3_-AHT group after treatment. Therefore, the sample size calculation was based on a 45% effective rate in the control group and a 75% effective rate in the O_3_-AHT group. Assuming a two-sided*a* = 0.05 and a statistical power of 0.9, the sample size was calculated to be 38 for each group. Considering a 15% loss to follow-up, the sample size was at least 44 in each group.

### 2.3. Therapeutic Method

All patients included in the study underwent routine examinations after admission to the hospital, including a blood routine examination, coagulation function, liver function, and kidney function.

#### 2.3.1. Pharmacological Management

Patients in both groups were given the same pharmacological management for 3 weeks. For insomnia management, patients were allowed to take 0.5–2 mg of estazolam if they found it extremely difficult to fall asleep. For pain management, patients with a VAS score <5 were given 60 mg of loxoprofen, with a maximum of 3 tablets per day. Patients with a VAS score of ≥5 were given tramadol 100 mg tablets, up to a maximum of 3 tablets per day.

#### 2.3.2. Ozonated Autohemotherapy

A special operator collected 100 ml of blood via the median cubital vein and injected it into a blood bag with an anticoagulant (10 ml of 3.8% sodium citrate). Blood was then mixed with a prepared 100 ml O_2_-O_3_ mixture by the ozone medical apparatus (Forefront Medical Equipment Co., Zibo, Shandong Province, China) for 3 minutes. Afterward, the ozonated autologous blood was transfused back into the patient within 10 minutes. Patients received ozonated autohemotherapy three times a week for 3 weeks. The concentration of ozone for each week was as follows: 20 *µ*g/ml in the first week, 30 *µ*g/ml in the second week, and 40 *µ*g/ml in the third week. 100 ml of the blood of patients in the control group was also collected without mixing with ozone, and then the sham infusion was performed. Only the special operator was aware of each patient's actual infusion condition.

### 2.4. Outcome Measurements

#### 2.4.1. Primary Outcomes

The insomnia severity index (ISI) was the primary outcome for evaluating the severity of insomnia in our study. This self-report questionnaire includes 7 items with a total score range of 0–28. Higher scores indicate more serious insomnia in the past two weeks.

The visual analogue scale (VAS) was the primary outcome for evaluating the severity of pain in our study. One end of a 10 cm straight line is written “painless,” and the other end is “the most severe pain.” According to the degree of pain they felt, patients made a mark at a certain point on the straight line to indicate the intensity of pain and psychological impact. The distance from the starting point to the mark is the amount of pain, 0–10 points. The higher the scores, the heavier the degree of pain.

#### 2.4.2. Secondary Outcomes

The Epworth sleepiness scale (ESS) is a questionnaire used to evaluate the degree of subjective somnolence. Patients recalled their recent experiences of drowsiness and scored the severity of drowsiness in different situations, such as sitting still, lying flat, watching TV, and driving. Then, we evaluated the severity of daytime drowsiness according to the total score. The sum score was calculated (range 0–24), with higher scores indicating more dangerous somnolence. We used ESS as the secondary outcome to evaluate sleep quality.

We utilized a polysomnography system (PolySmith, Neurotronics Co., FL, USA) to record the sleep data of patients at four time points (pretreatment, posttreatment, 1-monthfollow-up, and 6-month follow-up). At each time point, this instrument monitored the patient's sleep for 3 nights. Then, we collected the following data and took the following average: (1) total sleep time (TST)—sum of all asleep time in a night; (2) sleep onset latency (SOL)—time between going to bed and falling asleep; (3) wake after sleep onset (WASO)—sum of waking time in a night; (4) sleep efficiency (SE)—ratio of total sleep time to total time in bed; (5) number of awakenings (NOA)—times of awakening in a night.

The anxiety and preoccupation about sleep questionnaire (APSQ) consists of 10 self-reports of insomnia patients about their distress and anxiety about sleep, each with a score of 0–10. Individuals compared the consistency between these self-reports and their own conditions in the past three days. The total score is the sum of the 10 answers, with a range of 0–100. Higher scores indicate worse anxiety and preoccupation in the past three days.

The Beck depression inventory (BDI) contains 21 questions about the severity of depression in the past week, each with a score of 0–3. The total score ranges from 0–63, and higher scores indicate more severe depression.

The multidimensional fatigue inventory (MFI) assesses the following 5 aspects of fatigue: general fatigue, physical fatigue, reduced activity, reduced motivation, and mental fatigue. There are 20 questions in this questionnaire, each with a score range of 1–5. Higher scores indicate worse fatigue.

The treatment effect was assessed 6 months after treatment according to the following criteria: “remarkable”—the symptoms, especially insomnia and pain, almost disappeared, with good quality of life restored; “valid”—the symptoms, especially insomnia and pain, were relieved, with quality of life improved; “invalid”—no improvement in the symptoms, signs, or quality of life. The effective rate (%) = ((remarkable + valid)/*n*)*∗*100%.

#### 2.4.3. Adverse Complications

Possible complications, such as an allergic reaction, puncture point infection, hypotension, myocardial infarction, blood contamination, and abnormal blood potassium, were evaluated throughout the observation period.

### 2.5. Statistical Analysis

All data were analyzed using SPSS 26.0 (IBM Corporation, Armonk, NY, USA) and GraphPad Prism 5.0 (GraphPad Software, Inc., La Jolla, CA, USA). The data were presented as the mean ± SD or *n* (%). The independent-sample*t*-test was used for statistical analysis of the continuous data, and the chi-squared test was used for statistical analysis of the categorical data. The repeated-measures analysis of variance (ANOVA) test was used to compare data between the two groups before and after treatment. A *P*-value of <0.05 was considered to represent a statistically significant difference.

## 3. Results

### 3.1. Preoperative Patient Characteristics

**Figure 1:**
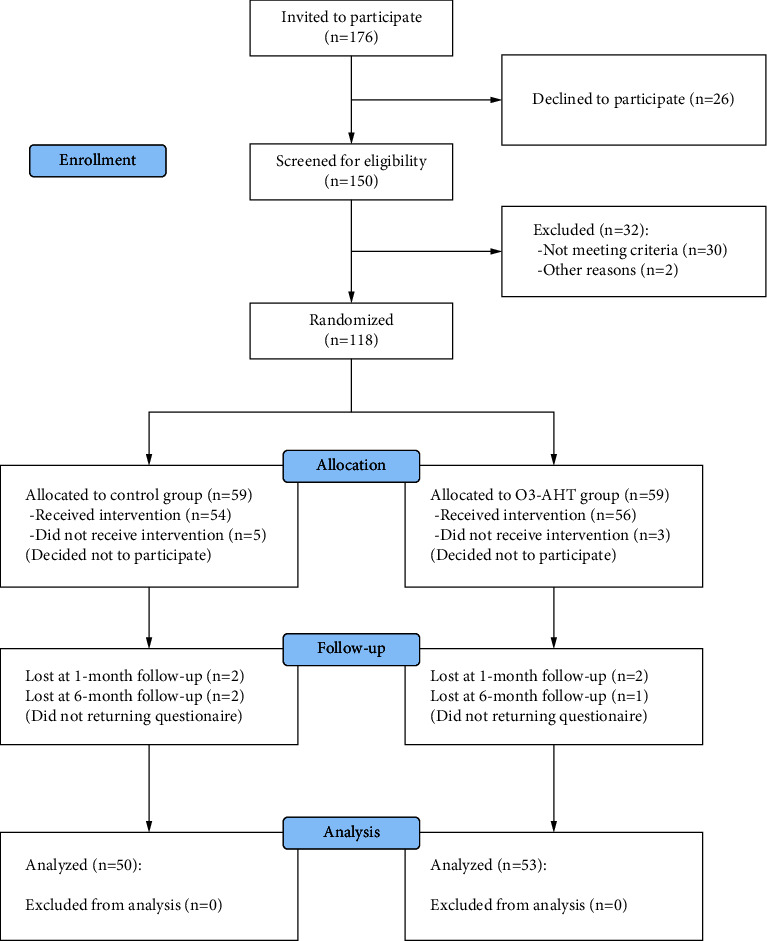
Consort flow diagram. O_3_-AHT, ozonated autohemotherapy.

We invited 176 patients to participate in this study, and 118 of them were randomized. Finally, 103 patients (50 in the control group and 53 in the O_3_-AHT group) completed each follow-up (Figure 1). No significant difference was found in the demographic and clinical characteristics between groups at baseline (*P* > 0.05) (Table 1).

### 3.2. Primary Outcomes

#### 3.2.1. Insomnia Severity Index

**Figure 2:**
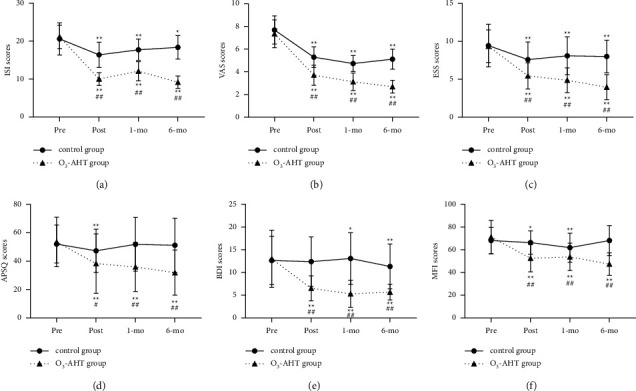
Comparison of ISI scores (a), VAS scores (b), ESS scores (c), APSQ scores (d), BDI scores (e), and MFI scores (f) in the two groups. ^∗^*p*  < 0.05; ^∗∗^*p*  < 0.01 versus the pretreatment within the same group. ^#^*p*  < 0.05; ^##^*p*  < 0.01 versus the control group. O_3_-AHT, ozonated autohemotherapy. ISI, insomnia severity index. VAS, visual analogue scale. ESS, Epworth sleepiness scale. APSQ, anxiety and preoccupation about sleep questionnaire. BDI, Beck depression inventory. MFI, multidimensional fatigue inventory. Pre, pretreatment. Post, posttreatment. 1-mo, 1-monthfollow-up. 6-mo, 6-monthfollow-up.

There was no significant difference between the two groups before treatment. In the control group, the ISI scores showed the lowest posttreatment and then gradually increased back. At each time point, the ISI scores of the O_3_-AHT group were significantly lower than pretreatment and showed the lowest at the 6-monthfollow-up. Meanwhile, the ISI score of the O_3_-AHT group was significantly lower than the control group at each time point after treatment (Figure 2(a)).

#### 3.2.2. Visual Analogue Scale

In the O_3_-AHT group, the VAS scores decreased gradually with time, and the VAS score was significantly lower than pretreatment at each time point. Similarly, compared with the baseline, an improvement in the VAS score was observed in the control group. However, at a 6-monthfollow-up, the VAS scores showed an increase compared with the 3-monthfollow-up. Between-group comparisons revealed that the VAS scores of the O_3_-AHT group were significantly lower than the control group at each time point after treatment (Figure 2(b)).

### 3.3. Secondary Outcomes

#### 3.3.1. Epworth Sleepiness Scale

Before treatment, there was no significant difference between the two groups. Compared with pretreatment, the ESS scores of the two groups decreased significantly at each time point. Between-groups comparisons revealed that the ESS scores of the control group were higher than those of the O_3_-AHT group at each time point (Figure 2(c)).

#### 3.3.2. Polysomnography

There was no significant difference between the two groups for TST, SOL, WASO, SE, and NOA before treatment. Between groups, comparisons revealed that, compared with the control group, data from the O3-AHT group were significantly improved at posttreatment, 1-monthfollow-up, and 6-monthfollow-up. Within-group comparisons revealed a significant improvement for TST, SOL, WASO, SE, and NOA of the O_3_-AHT group at each time point versus pretreatment. For TST and SOL, patients in the control group showed a significant improvement only at posttreatment. For WASO, except for the 1-monthfollow-up, patients in the control group had no improvement compared with pretreatment. For SE, patients in the control group gained significant improvement at posttreatment and the 6-monthfollow-up. However, for NOA, there was no difference between pretreatment and any time point after treatment in the control group (Table 2).

#### 3.3.3. Anxiety and Preoccupation about Sleep Questionnaire

The APSQ scores of the control and O_3_-AHT groups did not differ from each other before treatment. At the time of posttreatment, the APSQ scores of the O_3_-AHT group were lower than those of the control group. At the 1- and 6-monthfollow-ups, APSQ scores of the O_3_-AHT group showed a significant decrease compared with the control group. Within-group comparisons showed that the APSQ scores of the control group showed significant improvement only at posttreatment. APSQ scores of the O_3_-AHT group decreased significantly at each time point compared with pretreatment (Figure 2(d)).

#### 3.3.4. Beck Depression Inventory

The BDI scores of the control and O_3_-AHT groups did not differ from each other before treatment. At each time point after treatment, the BDI scores of patients in the control group were significantly higher than those in the O_3_-AHT group. Within-group comparisons revealed that in the control group, the BDI scores did not differ from pretreatment at the time point of posttreatment and even increased at the 1-monthfollow-up compared with pretreatment. At the 6-monthfollow-up, it showed a significant improvement compared with pretreatment. In the O_3_-AHT group, the BDI scores decreased significantly compared with pretreatment at each time point (Figure 2(e)).

#### 3.3.5. Multidimensional Fatigue Inventory

There was no difference between the MFI scores of the two groups before treatment. The MFI scores of patients in the control group were significantly higher than those in the O_3_-AHT group at each time point after treatment. Within-group comparisons showed that MFI scores of patients in the control group improved at posttreatment and 1-monthfollow-up but did not differ from pretreatment at 6-monthfollow-up. The MFI scores of the O_3_-AHT group decreased significantly compared with pretreatment at each time point (Figure 2(f)).

#### 3.3.6. The Effective Rate

The effective rate was 44% in the control group and 84.90% in the O_3_-AHT group 6 months after treatment (Table 3).

### 3.4. Adverse Complications

No adverse complications, such as an allergic reaction, puncture point infection, hypotension, myocardial infarction, blood contamination, or abnormal blood potassium, occurred in either group during the treatment and follow-up period. No patients withdrew from the treatment because of adverse complications.

## 4. Discussion

In our study, we comprehensively assessed the effects of O_3_-AHT on improving insomnia, ameliorating pain, and reducing negative emotions in patients with comorbid insomnia and MPS. Previous studies have generally regarded the improvement of insomnia symptoms as an additional outcome measure of O_3_-AHT in the treatment of painful diseases. In this trial, we put the assessment of sleep in a quite important position, and polysomnography was first utilized to record TST, SOL, WASO, SE, and NOA. Pharmacological therapy alone improved the ISI score to some extent after treatment, but from a long-term perspective, such improvement is inadequate and susceptible. Meanwhile, polysomnography results showed that pharmacological therapy alone only partially and temporarily improved symptoms of insomnia. By comparison, the advantages of O_3_-AHT combined with pharmacological therapy are reflected in more striking short-term improvements, a more comprehensive range of influence, and more sustained and incremental effects. The change in ESS and MFI scores also show that O_3_-AHT has a significant and lasting effect on improving insomnia-related somnolence and fatigue.

The improvement of pain in the two groups was consistent with expectations. The two groups both had therapeutic efficacies of different degrees. The VAS score was used to assess an individual's pain severity. The results of the VAS score show that the combined application of O_3_-AHT on the basis of pharmacological therapy can materially improve the pain relief effect. Patients with chronic myofascial pain are more likely to develop depression or anxiety [1, 2, 20]. Meanwhile, there is a close association between insomnia and negative emotions as well [21]. In our study, the APSQ and BDI scores of patients in the O_3_-AHT group showed a significant improvement, whereas there was no such improvement in patients treated by pharmacological therapy alone. These results are in line with our expectations.

Current studies believe that psychological, social, cultural, and biological factors will affect the pain perception of patients with MPS [22]. When chronic pain diseases such as MPS and insomnia occur together, the quality of life of patients will be greatly affected. A large number of studies have been carried out to explore the relationship between insomnia and pain, and there is no doubt that a bidirectional and mutually promoting relationship between insomnia and pain exists [4]. Pain can directly affect patients' sleep quality, and insomnia will reduce patients' pain perception threshold and increase their pain sensitivity. In addition, negative emotions, including depression, anxiety, and boredom, are important mediators connecting the relationship between pain and insomnia [23]. Pain, insomnia, and negative emotions form a triangular relationship wherein these three aggravate each other.

Contrary to popular knowledge, recent research suggests that sleep disorders may have a stronger influence on pain than vice versa [24]. Insomnia not only directly aggravates pain intensity but also affects the development of painful conditions. According to a large population-based study of Norwegian women who did not have fibromyalgia, there is a strong dose-dependent association between sleep disorders and the risk of fibromyalgia [25]. Another longitudinal cohort study in Norway analyzed the influence of insomnia at baseline on the risk for headache 11 years later and demonstrated a particularly strong association between insomnia and headache [26]. In conclusion, current evidence suggests that sleep disorders increase the risk of new-onset chronic pain in asymptomatic individuals and deteriorate the long-term prognosis of existing pain. When pain physicians treat headaches, musculoskeletal pain, or neuropathic pain, it is necessary to pay close attention to the accompanying insomnia. The essential role of insomnia in the onset and development of chronic pain is supposed to be of concern, which is why we regarded the assessment of sleep condition and insomnia severity as such an important component in our study.

Ozone therapy has been gradually applied in the field of medicine since its identification, although the process was circuitous and controversial [10]. Ozonated autohemotherapy (O_3_-AHT), the most mature and widespread form of ozone therapy in clinical practice, combines precollected blood with O_2_-O_3_ in vitro and then administers ozonated blood back to the patient. O_3_-AHT has been well received because it allows for a predetermined amount of blood to be taken and a precise concentration of O_2_-O_3_ to be infused [27]. It has been reported that O_3_-AHT can efficiently alleviate pain and improve functional scores in patients with low back pain, causing only occasional and slight adverse reactions [28].

Our study indicates the efficacy and safety of O_3_-AHT in the treatment of comorbid insomnia and MPS. This clinical effect may involve the antioxidant, anti-inflammatory, oxygen supply, and microcirculation mechanisms of ozone. Ozone has a strong antioxidant capacity. To be specific, after being dissolved in the aqueous component of plasma, ozone can initiate transient oxidative stress and an increased release of microparticles from blood cells [29]. In response, an endogenous cascade is induced, and then diverse bioactive substances are released. Ozone can react with polyunsaturated fatty acids (PUFA) and produce reactive oxygen species (ROS) and lipid ozonation products (LOPs) [30]. ROS and LOPs, which are considered significant in the ozone biotransformation, increase activation of nuclear factor erythroid 2-related factor 2 (Nrf2) and, thus, relieve pain [31]. The antioxidant capacity of ozone may play an important role in the alleviation of insomnia as well. By scavenging free radicals and limiting oxidative stress, ozone is beneficial for improving total antioxidant levels in the blood, which had been weakened by abnormal melatonin regulation [32]. Owing to these potential antioxidant functions, in our study, significant improvements in insomnia and pain were found.

Sleep restriction can elevate inflammatory markers, including interleukin-6 (IL-6), tumor necrosis factor (TNF), and C-reactive protein (CRP), which are considered to be related to the onset and mediation of pain [33, 34]. Ozone has the capacity to be anti-inflammatory and can decrease proinflammatory cytokines [35]. By anti-inflammatory effects, ozone may ameliorate insomnia severity and local inflammatory reaction. Meanwhile, the association between insomnia and increased pain experience may be weakening. Another famous inflammatory mediator—Prostaglandins (PG), especially PGE2, are not only important endogenous substances that regulate pain perception but have also been found to be elevated in patients with sleep disorders [36]. PG and its precursor, arachidonic acid, are classic PUFA, and as previously mentioned, ozone can react with PUFA after being dissolved in plasma. Therefore, it is reasonable to speculate whether ozone can alleviate insomnia and pain by affecting the metabolism of PG.

Ozone can improve the oxygen supply for tissues. O_3_-AHT can promote the activity of glycolysis and increase 2, 3-diphosphoglycerate (2, 3-DPG) in red blood cells [37]. 2, 3-DPG plays a key role in the rightward shift of the oxyhemoglobin dissociation curve and allows oxygen to be transferred to oxygen-starved tissues. On the one hand, increased oxygen supply to brain tissues can directly relieve insomnia. On the other hand, increased oxygen supply to localized tissues of pain regions, especially MTrPs, may alleviate hypoxia and hypermetabolism caused by continuous contraction of muscle fibers [38], so as to alleviate pain. In addition, ozone can improve the microcirculation in brain tissues and damage local tissues by increasing levels of vasodilators such as prostacyclin and nitric oxide [39]. The improvement in brain oxygen supply may be one of the reasons for the relief of anxiety and depression. To sum up, the effect of O_3_-AHT on insomnia and pain is complicated and comprehensive, and further studies are needed to clarify its specific mechanisms.

Consistent with previous studies [16, 17], we did not observe adverse complications caused by O_3_-AHT throughout the study. However, it is still necessary for researchers to carry out O_3_-AHT in a standardized procedure and actively notice any potential risk.

There are also the following limitations to our study: (1) the patients were recruited from a single centre; (2) double blinding was not used in this research; and (3) the patients were only followed for 6 months after treatment. we did not comprehensively analyse the interaction between insomnia and pain during treatment. Therefore, it is necessary to be cautious about the results of this study and the explanation for our findings.

## 5. Conclusions

In conclusion, our research demonstrated that ozonated autohemotherapy combined with pharmacological therapy was an effective and safe treatment for comorbid insomnia and myofascial pain syndrome. Compared with pharmacological therapy alone, this combination therapy can ameliorate insomnia, reduce pain intensity, improve related negative mood, and alleviate daily fatigue more significantly.

## Figures and Tables

**Table 1:** Patient characteristics before treatment.

Variables	Control group (*n* = 50)	O3-AHT group (*n* = 53)	*p* value^a^
Age (yr)	49.08 ± 8.35	48.15 ± 10.92	0.46
Gender			
Female	31 (62.0)	29 (54.7)	0.63
Male	19 (38.0)	24 (45.3)	
Marital status			
Single/divorced	4 (8.0)	5 (9.4)	0.80
Married/co-habiting	46 (92.0)	48 (90.6)	
Occupation			
Full time	18 (36.0)	24 (45.3)	0.18
Part time	8 (16.0)	11 (20.8)	
Sick leave	3 (6.0)	2 (3.7)	
Retired	21 (42.0)	16 (30.2)	
Height (cm)	165.52 ± 11.74	164.38 ± 13.28	0.64
Weight (kg)	63.26 ± 13.96	64.99 ± 12.59	0.51
BMI	19.70 ± 3.08	19.68 ± 2.80	0.87
Insomnia duration (*m*th)	19.18 ± 9.78	21.36 ± 7.78	0.22
Insomnia severity (ISI)	20.58 ± 4.24	21.13 ± 3.08	0.45
Pain duration (*m*th)	16.44 ± 7.84	17.49 ± 9.51	0.54
Pain severity (VAS)	7.69 ± 1.24	7.35 ± 1.22	0.84

Values are the means ± standard deviation and *n* (%). ^a^*t*-test or chi-square test as appropriate. O_3_-AHT, ozonated autohemotherapy; BMI, body mass index; ISI, insomnia severity index; VAS, visual analogue scale.

**Table 2:** Change of polysomnography data in the two groups.

	Pretreatment	Posttreatment	1-month	6-month	*p* value^a^		
					Pre vs. post	Pre vs. 1-month	Pre vs. 6-month
Total sleep time (min)							
Control group	355.60 ± 58.49	373.09 ± 58.74	360.58 ± 60.17	357.67 ± 63.87	<0.01	0.11	0.53
O_3_-AHT group	362.55 ± 58.45	415.20 ± 49.12	421.73 ± 51.86	428.62 ± 48.84	<0.01	<0.01	<0.01
*p* value^b^	0.55	<0.01	<0.01	<0.01			
Sleep onset latency (min)							
Control group	55.93 ± 18.15	49.55 ± 10.81	54.59 ± 14.59	53.43 ± 12.11	<0.01	0.24	0.02
O_3_-AHT group	54.64 ± 18.44	32.23 ± 15.58	31.85 ± 11.52	25.22 ± 9.32	<0.01	<0.01	<0.01
*p* value^b^	0.72	<0.01	<0.01	<0.01			
Wake after sleep onset (min)							
Control group	83.22 ± 34.24	80.15 ± 20.90	75.57 ± 24.23	78.88 ± 17.11	0.14	<0.01	0.04
O_3_-AHT group	86.90 ± 35.68	40.39 ± 13.04	39.76 ± 10.77	34.61 ± 12.94	<0.01	<0.01	<0.01
*p* value^b^	0.60	<0.01	<0.01	<0.01			
Sleep efficiency (%)^c^							
Control group	72.62 ± 8.89	75.26 ± 10.52	76.87 ± 8.13	73.96 ± 9.53	<0.01	<0.01	0.08
O_3_-AHT group	73.38 ± 12.18	87.59 ± 6.13	89.11 ± 6.12	91.45 ± 5.93	<0.01	<0.01	<0.01
*p* value^b^	0.72	<0.01	<0.01	<0.01			
Number of awakenings							
Control group	2.87 ± 1.85	2.88 ± 1.18	2.67 ± 1.23	2.79 ± 1.01	0.87	0.10	0.56
O_3_-AHT group	3.07 ± 1.95	1.52 ± 0.70	1.47 ± 0.97	1.40 ± 1.10	<0.01	<0.01	<0.01
*p* value^b^	0.60	<0.01	<0.01	<0.01			

Values are the mean ± standard deviation. ^a^repeated measures analysis of variance. ^b^independent-sample*t*-test. ^c^sleep efficiency = (total sleep time/total time in bed) × 100%. O_3_-AHT, ozonated autohemotherapy.

**Table 3:** Comparison of the effective rates in the two groups.

	Remarkable	Valid	Invalid	Effective rate
Control group (*n* = 50)	8	14	28	44.00%
O_3_-AHT group (*n* = 53)	26	19	8	84.90%^##^

^##^ *p* < 0.01 versus the control group. O_3_-AHT, ozonated autohemotherapy.

## Data Availability

The data used to support the findings of this study are available from the corresponding author upon request.
